# Positive Role of the MHC Class-I Antigen Presentation Regulator m04/gp34 of Murine Cytomegalovirus in Antiviral Protection by CD8 T Cells

**DOI:** 10.3389/fcimb.2020.00454

**Published:** 2020-08-26

**Authors:** Sara Becker, Annette Fink, Jürgen Podlech, Irina Giese, Julia K. Schmiedeke, Thomas Bukur, Matthias J. Reddehase, Niels A. Lemmermann

**Affiliations:** ^1^Institute for Virology, Research Center for Immunotherapy (FZI), University Medical Center of the Johannes Gutenberg-University of Mainz, Mainz, Germany; ^2^TRON - Translational Oncology, Medical Center of the Johannes Gutenberg-University Mainz gGmbH, Mainz, Germany

**Keywords:** adoptive cell transfer, antigen presentation, BAC mutagenesis, CD8 T cells, immune evasion, immunoevasin, next-generation sequencing (NGS), recombinant virus

## Abstract

Murine cytomegalovirus (mCMV) codes for MHC class-I trafficking modulators m04/gp34, m06/gp48, and m152/gp40. By interacting with the MHC class-Iα chain, these proteins disconnect peptide-loaded MHC class-I (pMHC-I) complexes from the constitutive vesicular flow to the cell surface. Based on the assumption that all three inhibit antigen presentation, and thus the recognition of infected cells by CD8 T cells, they were referred to as “immunoevasins.” Improved antigen presentation mediated by m04 in the presence of m152 after infection with deletion mutant mCMV-Δm06^W^, compared to mCMV-Δm04m06 expressing only m152, led us to propose renaming these molecules “viral regulators of antigen presentation” (vRAP) to account for both negative and positive functions. In accordance with a positive function, m04-pMHC-I complexes were found to be displayed on the cell surface, where they are primarily known as ligands for Ly49 family natural killer (NK) cell receptors. Besides the established role of m04 in NK cell silencing or activation, an anti-immunoevasive function by activation of CD8 T cells is conceivable, because the binding site of m04 to MHC class-Iα appears not to mask the peptide binding site for T-cell receptor recognition. However, functional evidence was based on mCMV-Δm06^W^, a virus of recently doubted authenticity. Here we show that mCMV-Δm06^W^ actually represents a mixture of an authentic *m06* deletion mutant and a mutant with an accidental additional deletion of a genome region encompassing also gene *m152*. Reanalysis of previously published experiments for the authentic mutant in the mixture confirms the previously concluded positive vRAP function of m04.

## Introduction

Human cytomegalovirus (hCMV) is the prototype member of the ß-subfamily of the herpesviruses [for an overview, see Davison et al. (2013)]. It is a clinically relevant pathogen leading to birth defects upon congenital infection, and it causes severe organ diseases in immunocompromised recipients of solid organ transplantation (SOT) and of hematopoietic cell transplantation (HCT) [for overviews, see Ho (2008), Boppana and Britt (2013), Emery et al. (2013), and Seo and Boeckh (2013)]. Despite expression of “immunoevasins” that limit the presentation of antigenic peptides to T cells [for reviews, see Wiertz et al. (1997), Hengel et al. (1998), Reddehase (2002), Doom and Hill (2008), Powers et al. (2008), Hansen and Bouvier (2009), and Berry et al. (2019)] unlimited viral spread and cytopathogenic tissue infection is prevented in the immunocompetent host, accompanied by the establishment of viral latency in certain cell types (Elder and Sinclair, 2019; Reddehase and Lemmermann, 2019). Reactivation to recurrent productive infection can occur when immune surveillance wanes due to immunocompromising conditions such as hematoablative leukemia therapy and immunosuppressive graft-vs.-host disease (GvHD) prophylaxis in HCT or immunosuppressive prophylaxis against graft rejection in SOT.

As experimental approaches and studies with recombinant viruses carrying targeted mutations for addressing mechanistic questions are not feasible in clinical investigations, the mouse model based on infection with murine cytomegalovirus (mCMV) has been developed as a versatile preclinical model. It has already provided “proof of principle” for basic aspects of viral pathogenesis and immune control, including cytoimmunotherapy with antiviral CD8 T cells in HCT recipients (Krmpotic et al., 2003; Reddehase, 2016; Reddehase and Lemmermann, 2018; Renzaho et al., 2020). Although the host species-specific CMVs differ in many genes involved in host adaptation, co-speciation of hosts and their respective CMVs has led to biological convergence in fundamental principles of virus-host interaction.

Three “immune evasion” proteins encoded by mCMV have been reported to bind to MHC class-I (MHC-I) molecules and to thereby disconnect them from the constitutive vesicular flow of trafficking to the cell surface in the MHC-I pathway of antigen processing and presentation to CD8 T cells (Lemmermann et al., 2012). These include the *m02-m16* gene family members m04/gp34 (Kleijnen et al., 1997) and m06/gp48 (Reusch et al., 1999), as well as the *m145* gene family member m152/gp40 (Ziegler et al., 1997, 2000; Fink et al., 2013). As far as analyzed, molecules of the *m02-m16* gene family share a β-sandwich immunoglobulin variable (Ig-V)-like fold (Berry et al., 2014; Sgourakis et al., 2014, 2015), whereas members of the *m145* gene family mimic the structure of MHC-I molecules, thus representing MHC-I-like virally encoded (MHC-Iv) glycoproteins (Wang et al., 2012). As shown by Fink et al. (2013), glycosylation of m152 is not required for m152 function, so that binding of the p36 isoform in the ER to nascent peptide-loaded MHC-I (pMHC-I) complexes and to the MHC-I-like ligand RAE-1 of the natural killer (NK) cell receptor NKG2D is sufficient for the dual role of m152 in inhibiting the activation of CD8 T cells and NK cells, respectively (Krmpotic et al., 2002). As concluded by implication from studies with immune evasion gene deletion mutants of mCMV (Wagner et al., 2002), evasion of antiviral CD8 T cells leads to enhanced and prolonged viral replication in recipients of experimental HCT with the consequence of an elevated latent viral genome load and increased risk of reactivation (Böhm et al., 2009). Immune evasion proteins reduce the efficacy of cytoimmunotherapy of mCMV infection by adoptive transfer of antiviral CD8 T cells (Krmpotic et al., 1999, 2002; Holtappels et al., 2004). More recently, mouse models of allogeneic HCT with immunogenetic donor-recipient mismatch in MHC-I or in minor histocompatibility loci revealed a decisive impact of viral immune evasion proteins on viral spread and lethal organ failure due to extensive histopathology (Gezinir et al., 2020; Holtappels et al., 2020). Thus, immune evasion is predictably of significant clinical relevance in HCT patients.

Regarding the molecular mechanisms, m152 mediates the retention of pMHC-I complexes in ER-Golgi intermediate/cis-Golgi compartments (Ziegler et al., 1997, 2000; Janßen et al., 2016). More recently, m152 has been identified to target the type I interferon response by binding to STING (Stempel et al., 2019). The closely related glycoproteins m04 and m06 compete for pMHC-I cargo by forming complexes and connect it to cellular adapter proteins (AP) of cargo sorting pathways via motifs in their cytosolic tails. Specifically, m06 contains a functional di-leucine motif that links the m06-pMHC-I complexes to AP-1A and AP-3A, eventually resulting in lysosomal disposal (Reusch et al., 2002). Recent work has shown that inactivation of the sorting motif by mutation does not prevent cell surface downmodulation of MHC-I molecules but inhibits the transition of m06-MHC-I complexes from early endosomes to late endosomes (Fink et al., 2019). In contrast, m04 contains a tyrosine-based AP-2 binding motif and escorts pMHC-I complexes to the cell surface (Kleijnen et al., 1997; Kavanagh et al., 2001; Lu et al., 2006; Fink et al., 2015) in association with the recently discovered viral protein MATp1. MATp1 turned out to be essential for m04-mediated MHC-I cell surface rescue resulting in silencing of NK cells via ligation of inhibitory Ly49 family NK cell receptors to overcome missing-self activation (Železnjak et al., 2019). Inactivation of the endocytic AP-2 motif was found to stabilize the complex at the cell surface, resulting in an enhanced NK cell silencing (Fink et al., 2015). The question remained if rescue of cell surface expression of pMHC-I complexes by m04 or m04-MATp1 also rescues recognition of infected cells by antiviral CD8 T cells. Although the m04 binding site to MHC-I has not been precisely mapped yet, structural data suggest that the peptide-binding platform is not masked (Berry et al., 2014), so that the TCR of CD8 T cells should still be able to recognize presented antigenic peptide.

When immune evasion proteins are expressed separately in viral mutants mCMV-Δm06m152 (selectively expressing m04), mCMV-Δm04m152 (selectively expressing m06), and mCMV-Δm04m06 (selectively expressing m152), inhibition of cell surface presentation of pMHC-I complexes ranked in the order of m04 << m06 < m152, with actually no notable inhibition by m04 [(Wagner et al., 2002; Holtappels et al., 2006); discussed in Lemmermann et al. (2012)]. This finding gave a first hint to raise doubt as to an “immune evasion” function of m04 with respect to CD8 T cells. Moreover, previous work revealed that m04 expressed in deletion mutant mCMV-Δm06^W^ (Wagner et al., 2002) relieves the inhibition mediated by co-expressed m152 as compared to mCMV-Δm04m06 expressing only m152 (Wagner et al., 2002; Holtappels et al., 2006). This was first shown on the basis of MHC-I cell surface levels detected cytofluorometrically (Wagner et al., 2002) and later extended functionally to target cell recognition (Holtappels et al., 2006; Pinto et al., 2006) and to *in vivo* protection by adoptively transferred antiviral CD8 T cells (Holtappels et al., 2006). This positive function of m04 prompted us to suggest the acronym “viral regulator of antigen presentation” (vRAP) to cover both positive and negative modulation of antigen presentation.

All conclusions based on virus mutant mCMV-Δm06^W^, expected to express normal levels of m152, needed to be drawn into question, because the authenticity of this virus was doubted based on the observation of an unexplained reduction of m152 protein expression that included all its glycosylation isoforms and that could not be reproduced with independently generated new mutants mCMV-Δm06^L1^ and mCMV-Δm06^L2^ (Fink et al., 2012, 2013; Lemmermann et al., 2012). As reduced expression of m152 relieves immune evasion, this alone might explain enhanced antigen presentation by cells infected with virus mutant mCMV-Δm06^W^ as compared to mutant mCMV-Δm04m06 expressing normal levels of m152. Misleadingly, genetic authenticity of mCMV-Δm06^W^ was suggested by qualitative detection of gene *m152* in liver tissue infected with mCMV-Δm06^W^ and analyzed by *in situ* hybridization (Holtappels et al., 2006). Accordingly, all attempts to explain the reduced expression by mutations in the *m152* gene coding region, the 3′ as well as 5′ untranslated regions, or the promoter region failed (unpublished own data).

As the problem not just casts doubt on our own previous conclusion on a positive vRAP function of m04 (Holtappels et al., 2006) but also might affect work of other groups who published data based on mCMV-Δm06^W^ (LoPiccolo et al., 2003; Pinto et al., 2006, 2007; Babic et al., 2010), we decided to compare the m152 expression-deficient mutant mCMV-Δm06^W^ and the m152 expression-sufficient mutant mCMV-Δm06^L^ by next-generation sequencing (NGS) of the full-length viral genomes purified from infectious virions. We identified two genomic variants within the pool of Δm06^W^ genomes: a small proportion of genomes with the correct, selective deletion of gene *m06* and a majority with an additional large deletion encompassing open reading frame (ORF) *m152* and spanning ORFs *m145*-*m158*. As implied by the growth curve of mutant mCMV-Δm06^W^ (Wagner et al., 2002), the large deletion does not lead to attenuated growth in cell culture and therefore is maintained in purified virus stocks. Mapping of the large deletion allowed us to design specific probes for *in situ* hybridization distinguishing between the correct deletion mutant mCMV-Δm06 and the wrong mutant with the additional off-target site deletion. Reanalysis of stored tissue specimens from key experiments of the original work (Holtappels et al., 2006) confirmed a positive vRAP function of m04 in liver cells infected with the minority fraction of the correct *m06* gene deletion mutant present in the mixed pool.

## Materials and Methods

### Cells and Viruses

High titer virus stocks of mCMV^Smith^ sequence-derived mCMV-WT.BAC (MW97.01, Wagner et al., 1999), mCMV-Δm06^W^ (Wagner et al., 2002), and mCMV-Δm06^L^ (Fink et al., 2012), were prepared from infected murine embryonic fibroblasts (MEF) according to standard procedures (Podlech et al., 2002; Lemmermann et al., 2010).

### Purification of Viral DNA From High Titer Virus Stocks

Viral DNA corresponding to 1 × 10^7^ plaque forming units (PFU) was purified from a high titer virus stock by using Roche High Pure Viral Nucleic Acid Kit (Roche) following the manufacturer's instruction. Viral DNA was eluted in 50 μL of elution buffer and stored at 4°C until use for DNA library preparation or for PCR.

### NEBNext Ultra Library Preparation

In total, eight DNA libraries were prepared using NEBNext Ultra II DNA Library Prep Kit for Illumina (NEB). As the first step, 100 ng of viral DNA was sheared to an average size of around 250 bp by ultrasonication. Fragments were checked via capillary gel electrophoresis, end-repaired, A-tailed, and Illumina-specific barcoded adapters were ligated. These adapters also serve as annealing regions for the amplification primers during a PCR. The libraries were checked for quantity and quality using Bioanalyzer2100 (Agilent) and Qubit 3.0 Fluorometer (Thermo Fisher Scientific). Subsequently, the libraries were normalized to 10 nM and pooled to be equimolar before sequencing.

### Next-Generation Sequencing on Illumina MiSeq

Sequencing templates were immobilized on a flow cell surface designed to present the DNA in a manner that facilitates access to enzymes while ensuring high stability of surface-bound template and low non-specific binding of fluorescent-labeled nucleotides. Solid-phase amplification creates up to 1,000 identical copies of each single template molecule in close proximity. Sequencing by synthesis (SBS) technology uses four fluorescent-labeled nucleotides to sequence the tens of millions of clusters on the flow cell surface in parallel. During each sequencing cycle, a single labeled deoxynucleotide triphosphate (dNTP) is added to the nucleic acid chain. The nucleotide label serves as a terminator for the extension. So, after each dNTP incorporation, the fluorescent dye is imaged to identify the base and is then enzymatically cleaved to allow incorporation of the next nucleotide. Since all four reversible terminator-bound dNTPs (A, C, T, G) are present as single separate molecules, natural competition minimizes incorporation bias. Base calls are made directly from signal intensity measurements during each cycle. Here, libraries have been sequenced in four batches on an Illumina MiSeq Nano Flow cell (2 × 150 bp, paired-end) with an average output of 1 million clusters passing filter. Each batch contained a pool of two equimolar pooled libraries.

### Analysis of Sequencing Data

Sequencing yielded between 1.40 and 1.91 million paired-end reads (mean: 1.59 million read pairs). Reads were trimmed of adapter sequences, filtered for read length (minimum of 15 bases by default), and overlapping read fragments were error corrected using Fastp (version 0.19.4; Chen et al., 2018). On average, 1.58 million read pairs were left for alignment (minimum: 1.39 million, maximum: 1.89 million). The reads were aligned using the Smith-Waterman alignment method implemented in Novoalign (version 3.09.01; http://www.novocraft.com/products/novoalign/) against the mCMV reference (RefSeq: NC_004065.1, INSDC: U68299.1). Alignments resulted in a mean coverage between 199.1 and 441.6 per sample on the reference. Single nucleotide variations (SNVs) and small insertions and deletions (INDELs) were identified using Fisher's Exact Test implemented in VarScan 2 (http://varscan.sourceforge.net; Koboldt et al., 2012). SNVs and INDELs were reported with a *P*-value below 0.01 for somatic calls. We inspected larger variations from the reference genome visually using the Integrative Genomics Viewer (version 2.4.13) (Robinson et al., 2011).

### Validation of Deletion in the *m145* Region

To validate a possible deletion within the *m145* region, PCR was performed using the HotStarTaq DNA Polymerase (Qiagen) and oligonucleotides (*m145*_flank_for: CACGACAGACATACAGAG, *m145*_flank_rev: GCAGACTCTGAGGACCGG) with the following profile: 95°C 15 min; (95°C 15 s; 50°C 60 s; 72°C 90 s) × 35; 72°C 10 min. For the amplification of the potential 13 kbp-long inconsistent region, the LongRange PCR Kit (Qiagen) was used and PCR was performed with oligonucleotides (large_del_flk_for: GGTGAGGGGATTATGTCCTG, large_del_flk_rev: TGGTGGTGCCCTATCCTTAC) under the following conditions: 93°C 3 min; (93°C 15 s; 50°C 30 s; 68°C 13 min) × 10; (93°C 15 s; 50°C 30 s; 68°C 13 min with +20 s elongation for each cycle) × 28. The PCR products were separated on TAE agarose gel and bands of interest were cut out. DNA was purified with the QIAquick Gel Extraction Kit (Qiagen) and the PCR product was sequenced by Eurofins GATC services (Freiburg) using Sanger-Seq1_for: AGGCACGTAGCGAGGATGTC, Sanger-Seq2_for: GATGACGTACTCTCCCTG, Sanger-Seq3_for: GCGGACGACCTCGTTGAG, Sanger-Seq4_for: CGTTAACCGGGCTGCATCC.

### Detection and Distinction of mCMV Variants by *in situ* DNA-DNA Hybridization

Sequence-specific two-color DNA-DNA *in situ* hybridization (2C-ISH) was used to detect and quantitate infected cells in liver tissue sections. For distinguishing cells infected with different virus variants in a mixture, variant-specific ISH probes were generated and labeled by PCR with digoxigenin-11–dUTP or fluorescein-12-dUTP, followed by peroxidase-conjugated anti-digoxigenin antibody or alkaline phosphatase-conjugated anti-fluorescein antibody for black (DAB-nickel) and red (Fuchsin) color staining, respectively (Lemmermann et al., 2010). Primers for probe synthesis were as follows: (mCMV *m152*-P), *m152*-P_for: AGTTGATGTAGACCAGGCGATAC, *m152*-P_rev: GCTATCACCTACTTGCTCCTCTCG. (mCMV *M55/*gB-P), *M55-P*_for: AAGCTTGCACGTCGTAGGTAAATTGC, *M55*-P_rev: CAGGATCCTCGTCTCTCGAGCTGGTACG. (mCMV BAC-P), BAC-P_for: CACTGTTCCACTTGTATCG, *BAC*-P_rev: CATGCAGCTCCCGGAGACG.

### Detection and Distinction of mCMV Variants by Immunohistochemistry

As a more sensitive alternative to 2C-ISH, two-color immunohistochemistry (2C-IHC) was used to detect and quantitate infected cells in liver tissue sections. For distinguishing cells infected with different virus variants in a mixture, antibodies specific for differentially expressed viral proteins were labeled either with biotinylated second antibody and ABC-peroxidase for black (DAB-nickel) staining or with alkaline phosphatase conjugated second antibody for red (Fuchsin) staining, essentially as described (Lemmermann et al., 2010). Monoclonal rat antibody (clone M3D10) was used as the primary antibody in the red staining of m152 (Ziegler et al., 2000).

### Adoptive Transfer of Antiviral CD8 T Cells

Stored tissue specimens from previously published adoptive cell transfer experiments (Holtappels et al., 2006) using CD8 T cells of cytolytic T-lymphocyte (CTL) lines specific for antigenic peptides derived from mCMV protein M45, namely peptide 507-VGPALGRGL-515 presented by the MHC-I molecule D^d^ (epitope M45-D^d^) and peptide 985-HGIRNASFI-993 presented by D^b^ (epitope M45-D^b^) (Gold et al., 2002; Holtappels et al., 2009) were reanalyzed by 2C-ISH and 2C-IHC, respectively.

## Results

### NGS Reveals a Large Deletion in BAC-Derived mCMV-Δm06^W^ Genomes

**Figure 1:**
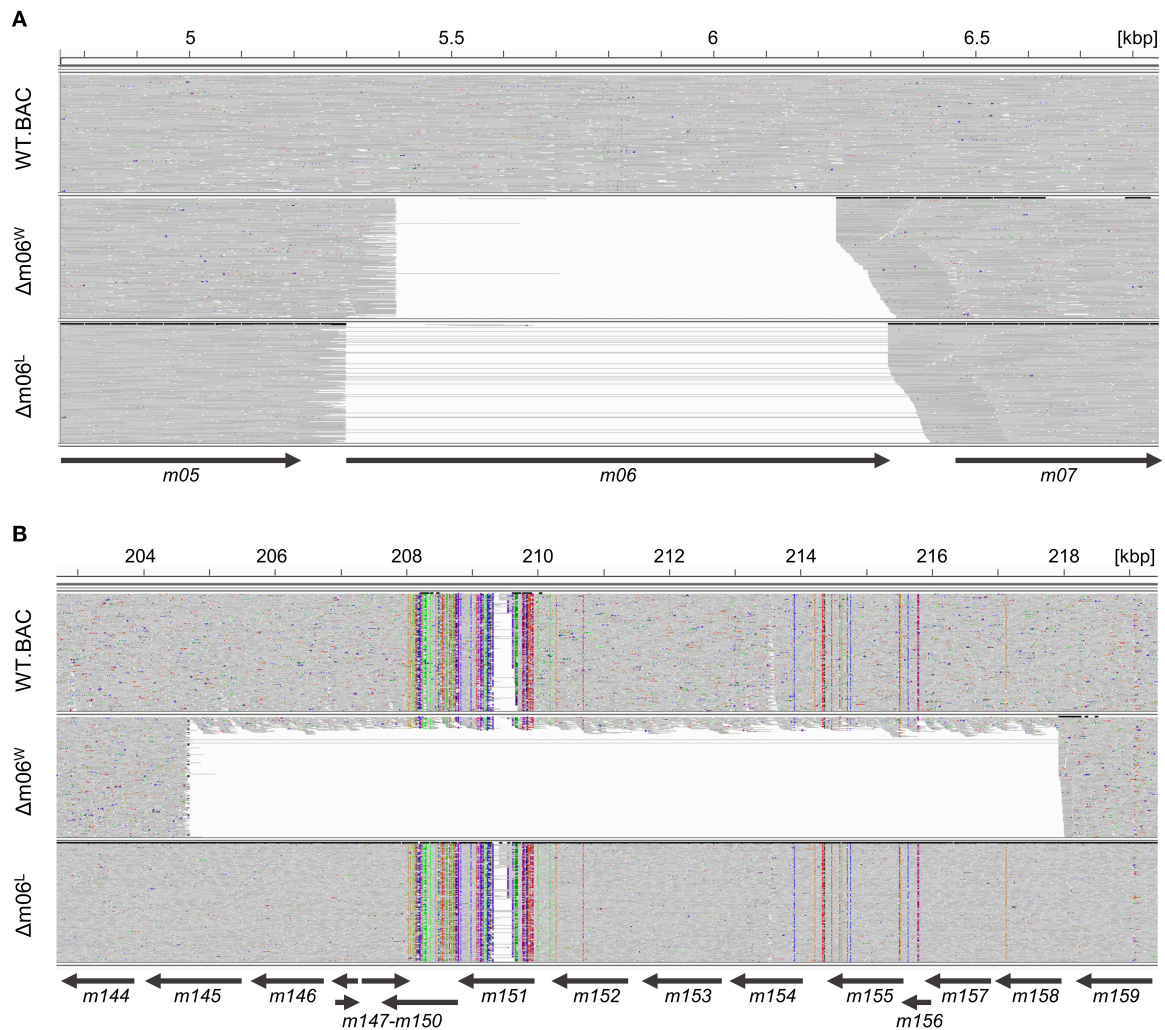
Comparison of Illumina-sequenced viral genomes. Viral DNA was purified from stocks of viruses mCMV-WT.BAC, mCMV-Δm06^W^, and mCMV-Δm06^L^, sequenced on Illumina MiSeq, and aligned to the mCMV WT reference sequence (RefSeq: NC_004065.1, INSDC: U68299.1). The Integrative Genomics Viewer was used for visualization. Gray areas indicate matches to the reference sequence, whereas colored spots indicate SNVs or INDELs. Regions with no successful alignment to the reference sequence are left white. Arrows represent the positions of the indicated ORFs. **(A)** Comparison of the viral genomes at 4.8–6.8 kbp. **(B)** Comparison of the viral genomes at 203–219 kbp.

To investigate the reason for the unexpectedly diminished m152 expression in cells infected with mCMV-Δm06^W^ (Fink et al., 2012, 2013; Lemmermann et al., 2012) we tested the genetic authenticity of this mutant in comparison to mCMV-WT.BAC and the independently generated mutant mCMV-Δm06^L^ (Fink et al., 2012) by full-length genome next-generation sequencing (NGS) of purified virion DNA. First, we verified the deletion of gene *m06* in mCMV-Δm06^W^ (nts. 5,396–6,236; GeneBank U68299.1; Rawlinson et al. (1996) and mCMV-Δm06^L^ (nts. 5,301–6,334) (Figure 1A). Full-length genome comparison of mCMV-Δm06^L^ and mCMV-WT.BAC revealed no unexpected differences between both genomes. In contrast, the sequencing revealed a large deletion in mCMV-Δm06^W^ (Figure 1B) spanning ≈13 kbp (nts. 204,704–217,934) encompassing 14 ORFs from *m145* to *m158*, many of which code for MHC-Iv glycoproteins that have been associated with modulation of host innate and/or adaptive immunity.

Besides m152, the object of our investigation here, more prominent examples affected by the deletion include m145 and m155 that downmodulate NKG2D ligands MULT-1 (Krmpotic et al., 2005) and H60 (Hasan et al., 2005), respectively, the activatory ligand m157 of the Ly49H NK cell subset that confers genetic resistance to mCMV by controlling early virus replication in C57BL/6 (haplotype *H-2*^*b*^) mice (Arase et al., 2002; Scalzo et al., 2003; Voigt et al., 2003; Bubić et al., 2004; Fodil-Cornu et al., 2008), m154 that reduces the cell-surface expression of the SLAM family member CD48 (Zarama et al., 2014) and mediates broad-spectrum immune evasion by perturbing the adaptor protein-1 compartment (Strazic Geljic et al., 2020), and m147.5 that downmodulates the co-stimulatory molecule CD86/B7-2 on antigen-presenting cells (Loewendorf et al., 2004). So, obviously, a phenotype of mutant virus mCMV-Δm06^W^ cannot be attributed with any certainty to the deletion of m06.

Notably, however, a small proportion of the annotated reads aligned within the deleted *m145* region, indicating that the mCMV-Δm06^W^ virus stock harbors a mixture of at least two different sets of viral genomes. The ratio of the two genomes can be roughly estimated from the mean coverage within the deletion (15.14 reads per base) to the mean coverage of the entire sequence except the deletion (209.6 reads per base). This estimate indicates that ≈10% of the genomes represent a correct m06 deletion mutant.

**Figure 2:**
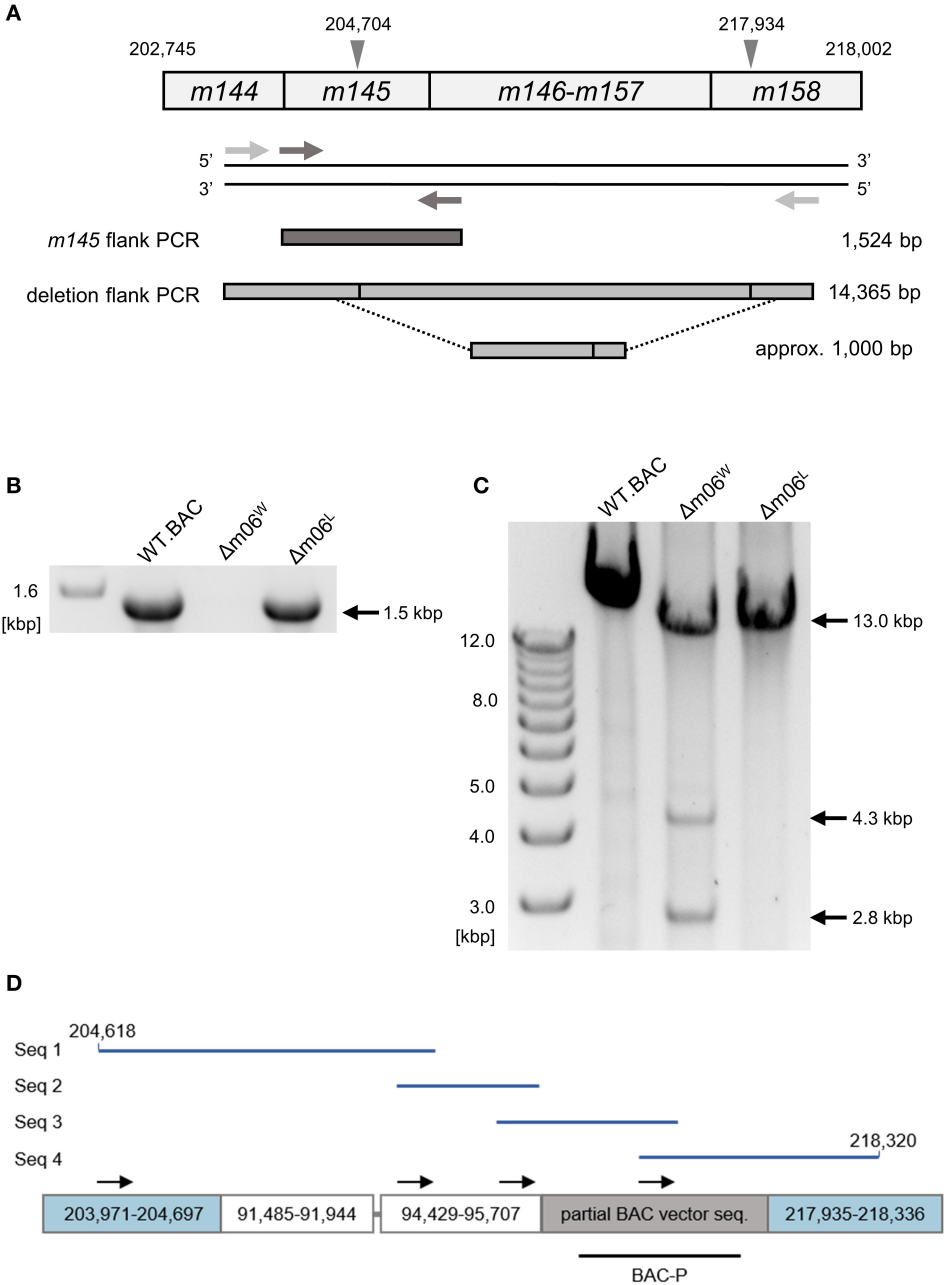
Mapping of the deletion in mCMV-Δm06^W^. **(A)** Map of the primer design. Gray arrowheads demarcate the suspected deletion in the *m145* gene family, numbers indicate nucleotide positions in the mCMV genome. Light gray arrows represent primers flanking the site of deletion, dark gray arrows represent primers flanking the *m145* gene. Expected PCR product sizes are shown on the right side. **(B)** PCR products of the *m145*-flank PCR on an agarose gel. **(C)** PCR products of the deletion-flank PCR on an agarose gel. Purified DNA from mCMV-WT.BAC, mCMV-Δm06^W^, and mCMV-Δm06^L^ virus stocks was used as template in both **(B,C)**. **(D)** Sanger sequencing of the 4.3 kbp-sized deletion-flank PCR product in mCMV-Δm06^W^. The PCR product was gel-purified and used for Sanger sequencing. In total, four primers were used. Black arrows indicate their map positions. Sequences (Seq 1–4, blue lines) were aligned with the mCMV genome and the matching regions are displayed by the boxes. The gray linker between the second and third box represents a short (11 bp) unmatched region. The lower black bar represents the ISH probe used to identify the “large deletion” mutant mCMV-Δm06m145-158 (probe BAC-P). Numbers indicate nucleotide positions in the mCMV genome.

To confirm and more precisely map the deletion of the *m145-m158* region, we used the primer pair *m145*_flank_for and *m145*_flank_rev for amplifying ORF *m145* (Figure 2A). With the chosen assay sensitivity, *m145* could be amplified from purified mCMV-WT.BAC and mCMV-Δm06^L^ genomes, but not from mCMV-Δm06^W^ genomes (Figure 2B). The absence of *m145* amplification in mCMV-Δm06^W^ indicated a deletion in the *m145-m158* region. To further test if the missing 13 kbp represent a full deletion or an undesired recombination of viral or cellular DNA fragments, we tried to amplify a fragment spanning the potential deletion site with the primer pair large_del_flk_for and large_del_flk_rev. In case of a full deletion, a ≈1 kbp fragment was expected. However, we were not able to amplify such a fragment. Therefore, we used an optimized protocol for amplification of fragments up to 15 kbp, and this resulted in three fragments with sizes of ~2.8, 4.3, and 13 kbp (Figure 2C). The 2.8 kbp fragment was identified by sequencing as a fragment of the *m145-m146* region, and the 13 kbp fragment was identified as the full-length *m145-m158* region. Both fragments were derived from the small proportion of viral genomes in the virus stock. The 4.3 kbp PCR product, however, was identified as an irregular, disjointed 4,295 bp fragment of the mCMV genome containing parts of ORFs *M57* and *M58* (nts. 91,485–91,944) and a non-coding intergenic sequence (nts. 94,429–95,707). The following 1,429 nts proved to be a truncated BAC vector sequence of the parental mCMV-WT.BAC plasmid C3X (Wagner et al., 1999) (Figure 2D).

Collectively, these data thus indicate that during the generation of mCMV-Δm06^W^ virions an unwanted recombination event must have taken place at some step. This resulted in a deletion of gene region *m145-m158* in the majority of the viral genomes. Apparently, the accidental “large deletion” mutant mCMV-Δm06m145-158 was not lost from the mixture during virus propagation, which is in accordance with the growth curve shown in the original report (Wagner et al., 2002) and explained by the fact that the array of deleted genes is known to be non-essential for virus growth in cell culture.

### Lack of *in vivo* Selection Against the “Large Deletion” Mutant in Immunocompromised Mice

Based on these new data we have now learned in retrospect that our *in vivo* experiments performed previously with mCMV-Δm06^W^ (Holtappels et al., 2006) were unknowingly performed as co-infection experiments with a mixture of correct mutant mCMV-Δm06 (≈10%) expressing m04 and m152, and a mutant mCMV-Δm06m145-158 with accidental co-deletion affecting m152 (≈90%), thus functionally resembling a Δm06m152 mutant. From previous work we have great experience in analyzing intended co-infections with virus variants by 2C-ISH performed with specific DNA probes to distinguish the variants in host tissues (Grzimek et al., 1999; Holtappels et al., 2004; Cicin-Sain et al., 2005). As a bottom-line message from these studies, spread- and growth-competent variants rarely co-infect tissue cells but establish separate, clonal foci of infection (Grzimek et al., 1999; Holtappels et al., 2004), whereas a requirement for functional complementation selects for co-infected cells (Cicin-Sain et al., 2005).

**Figure 3:**
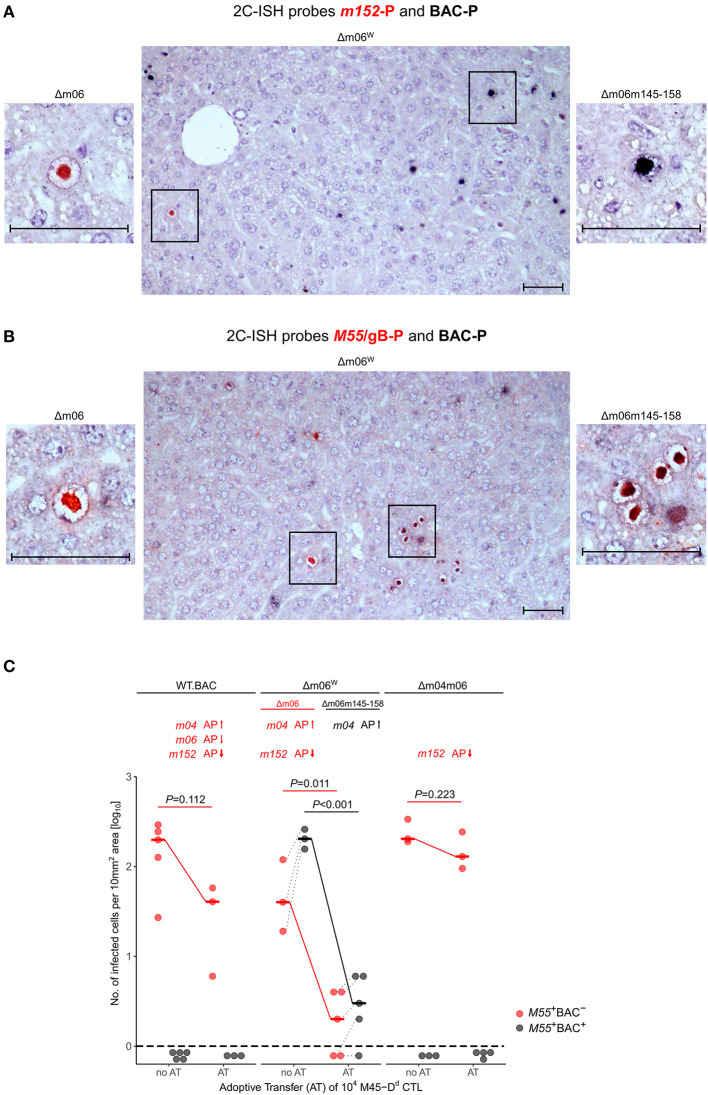
2C-ISH analysis of liver infection. Reanalysis of stored liver specimens from a previously performed experiment [lung virus titers shown in Holtappels et al. (2006), Figure 8B]. BALB/c mice were immunocompromised by γ-irradiation (6.5 Gy) and infected at one footpad. Liver tissue sections were taken on day 12 after infection **(A,B)** Virus spread in liver tissue. Infection was performed with 10^5^ PFU of mCMV-Δm06^W^, now identified to represent a mixture of correct virus mCMV-Δm06 and a “large deletion” mutant mCMV-Δm06m145-158 that includes deletion of the *m152* gene. **(A)** 2C-ISH performed with probe *m152*-P (red staining) specific for mCMV-Δm06 and probe BAC-P (black staining) specific for mCMV-Δm06m145-158. (Center panel) overview image showing foci of infection for both viruses in the mixture representing mCMV-Δm06^W^. (Left panel) red-stained mCMV-Δm06-infected cell resolved to greater detail. (Right panel) black-stained mCMV-Δm06m145-158-infected cell resolved to greater detail. **(B)** 2C-ISH performed with probe *M55*/gB (red staining) specific for both viruses and probe BAC-P (black staining) specific for mCMV-Δm06m145-158. (Center panel) overview image showing foci of infection for both viruses in the mixture representing mCMV-Δm06^W^. (Left panel) red-stained mCMV-Δm06-infected cell resolved to greater detail. (Right panel) red-black speckled cells infected with mCMV-Δm06m145-158, resolved to greater detail. Frames in the overview images indicate tissue section areas resolved to greater detail in the left and right images. Note the stained CMV-typical inclusion bodies in the nuclei of infected hepatocytes. Counterstaining was performed with hematoxylin. Bar markers: 50 μm. **(C)** vRAP expression-dependent control of liver tissue infection with the indicated viruses on day 12 after adoptive transfer of 10^4^ antiviral CD8 T cells specific for the viral epitope M45-D^d^. 2C-ISH was performed with probes *M55/gB*-P (red symbols) and BAC-P (black symbols). (no AT) no adoptive transfer. (AT) adoptive transfer. vRAPs actually expressed by the viruses as well as their proposed impact on antigen presentation (AP, arrows up or down) are indicated. Data represent counts of infected liver cells in representative 10 mm^2^ tissue section areas. The dashed line indicates the detection limit of the assay, which is one infected cell per selected counting area. Symbols represent mice tested individually. Median values are marked and connected for the groups “no AT” and “AT” to highlight the strength of antiviral control. For statistical analysis, data were log-transformed and *P*-values were calculated by using the two-sided unpaired *t*-test with Welch's correction of unequal variances. *P* < 0.05 indicates statistical significance of the difference between “no AT” and “AT” groups. Linked data are connected by dotted lines, which reveals a correlation between the numbers of cells infected with the correct mutant mCMV-Δm06 and the “large deletion” mutant mCMV-Δm06m145-158 after infection with mCMV-Δm06^W^.

By using hybridization probes specific for the correct Δm06 mutant carrying gene *m152* (probe *m152*-P, red intranuclear staining) and for mutant mCMV-Δm06m145-158 that contains BAC vector sequence (probe BAC-P, black intranuclear staining), we reanalyzed stored liver tissue from a previously published experiment (Holtappels et al., 2006), in which immunocompromised, total-body γ-irradiated BALB/c (haplotype *H-2*^*d*^) mice had been infected with mCMV-Δm06^W^. Infected liver cells, detected on day 12 after infection, mostly represent hepatocytes, but also non-parenchymal liver cells such as liver sinusoidal endothelial cells (LSEC) and liver macrophages, known as Kupffer cells (Sacher et al., 2008; Lemmermann et al., 2015). Probes *m152*-P and BAC-P distinguished between liver cells infected with the correct mutant mCMV-Δm06 (clean red staining) and those infected with mutant mCMV-Δm06m145-158 (clean black staining) (Figure 3A). Notably, cells infected with either virus remained spatially separate in clonal foci of infection, and dually-infected liver cells were not found. Cells infected with mCMV-Δm06m145-158 prevailed in numbers. Lack of red staining with *m152*-P in cells stained black with BAC-P makes absence of gene *m152* in the “large deletion” mutant visible. As an alternative detection strategy, probe *M55*/gB-P was used to detect all infected cells by red staining, and probe BAC-P was again used to identify cells infected with mCMV-Δm06m145-158 by black staining (Figure 3B). In this case, cells infected with the correct Δm06 mutant can again be identified by clean red staining, whereas cells infected with mCMV-Δm06m145-158 show up by a speckled black-red staining. Again, foci of infection were found to be spatially separate, and cells infected with mCMV-Δm06m145-158 prevailed in numbers. Note that the deletion of genes involved in innate immune control has no phenotype in mice immunodepleted by γ-irradiation. Combined, the data show independent growth of both mutants in liver tissue of immunocompromised mice and do not indicate an *in vivo* growth attenuation of mCMV-Δm06m145-158 in the absence of innate and adaptive immune control.

### Verification of m04 as a Positive vRAP by Transfer of CTL Specific for Epitope M45-D^d^

As the viruses differ in the expression of vRAPs that regulate antigen presentation to CD8 T cells, a selection pressure was introduced into the immunocompromised BALB/c mice by adoptive transfer (AT) of 10^4^ antiviral CD8 T cells of a CTL line specific for the antigenic peptide M45_507_-VGPALGRGL-_515_ that is presented by the MHC-I molecule D^d^, briefly epitope M45-D^d^ (Holtappels et al., 2006). Stored tissue specimens from this published experiment were reanalyzed to either confirm or disprove the previous conclusions. The reanalysis was performed by 2C-ISH of liver tissue of mice infected with viruses mCMV-WT.BAC, mCMV-Δm06^W^, and mCMV-Δm04m06 with hybridization probes *M55*/gB-P (red staining) for detection of all infected cells and BAC-P (black staining) selectively for detection of cells infected with mutant virus mCMV-Δm06m145-158, which is only present in the virus mixture of mCMV-Δm06^W^ (Figure 3C). Confirming previous data (Holtappels et al., 2006), control of mCMV-WT.BAC expressing all three vRAPs and of mCMV-Δm04m06 expressing only the strongest inhibitory vRAP m152 was poor and did not reach statistical significance. Not unexpectedly, mCMV-Δm06m145-158 within mCMV-Δm06^W^ was controlled most efficiently and with high statistical significance, because it lacks both inhibitory vRAPs m06 as well as m152, so that antigen presentation to the transferred CD8 T cells is optimal. For conclusions on a predicted function of m04 as a positive vRAP counteracting the negative vRAP m152, the analysis must be restricted to cells infected with the minority fraction of correct mCMV-Δm06 in the virus mixture of mCMV-Δm06^W^. Importantly, control of correct mutant mCMV-Δm06 that expresses m04 along with m152 was significantly more efficient than was control of mCMV-Δm04m06 expressing m152 only.

### Confirmation of m04 as a Positive vRAP by Transfer of CTL Specific for Epitope M45-D^b^

The question remained if a positive vRAP function of m04 with respect to virus control by CD8 T cells may be an exception that applies only to antigen presentation by the MHC-I molecule D^d^, a question raised because MATp1-dependent cell surface expression was found to be most prominent for m04-D^d^ and m04-K^b^ complexes and less for other MHC-I alleles as far as analyzed (Železnjak et al., 2019). We have previously intensely studied the antigenic peptide M45_985_-HGIRNASFI-_993_ that is presented by the MHC-I molecule D^b^, briefly epitope M45-D^b^ (Gold et al., 2002), because it is so far the only known epitope so poorly presented in the presence of negative vRAPs that specific CTL completely fail to protect immunocompromised C57BL/6 mice upon adoptive cell transfer, unless vRAP m152 is deleted (Holtappels et al., 2004). A comparison between M45-D^d^ and M45-D^b^ is particularly appealing, because both peptides are processed from the same protein, which is a cell death inhibitor (Brune et al., 2001; Daley-Bauer et al., 2017), so that differences cannot be attributed to properties of the protein. As we have shown previously, poor protection by M45-D^b^ CTL in C57BL/6 mice compared to M45-D^d^ CTL in BALB/c mice relates to a very low processing efficacy yielding only few peptide molecules per infected cell, so that negative vRAPs can easily prevent cell surface presentation and thus protection (Holtappels et al., 2009).

**Figure 4:**
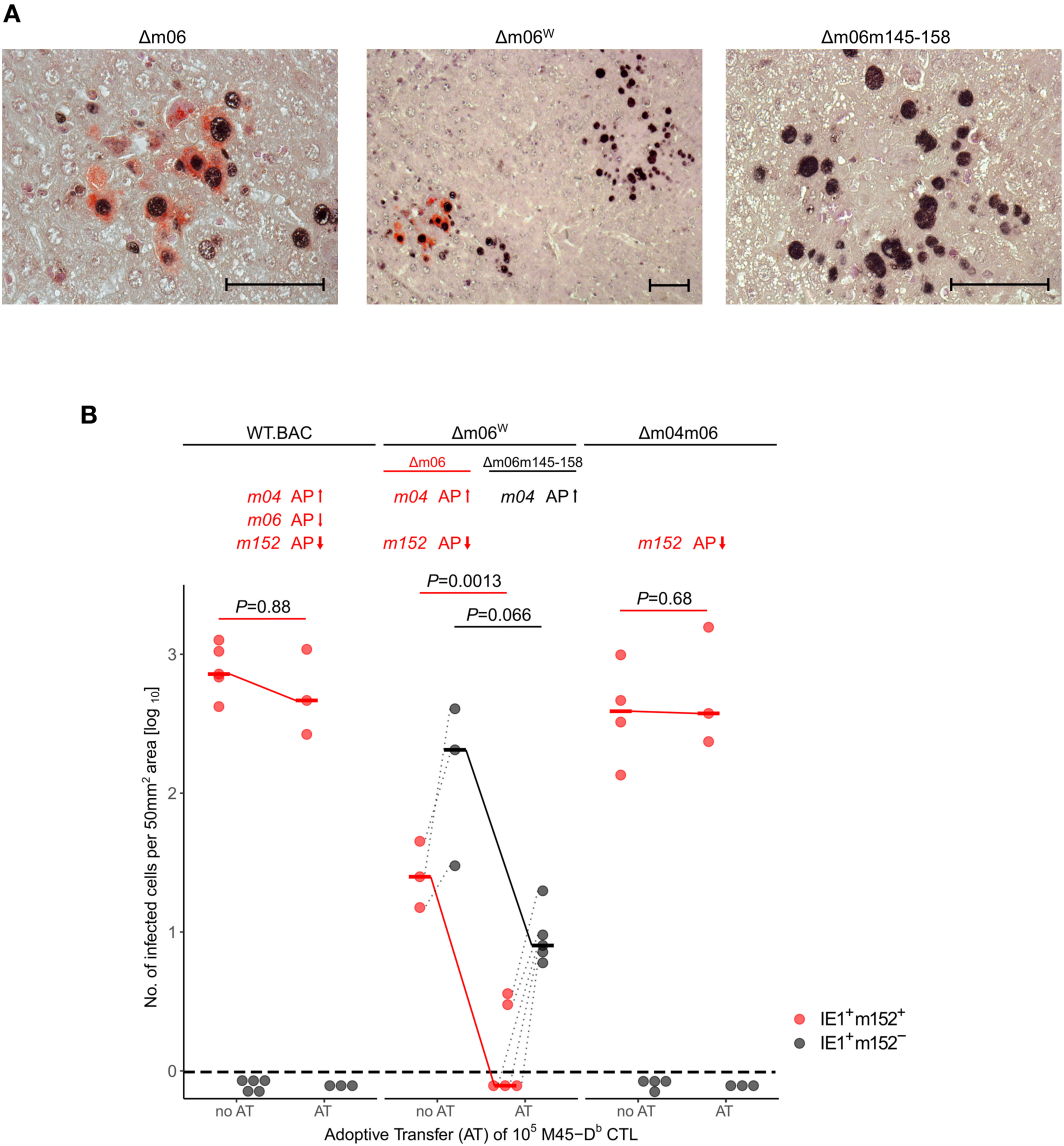
2C-IHC analysis of liver infection. Reanalysis of stored liver specimens from a previously performed experiment [lung virus titers shown in Holtappels et al. (2006), Figure 8A]. C57BL/6 mice were immunocompromised by γ-irradiation (7.5 Gy) and infected at one footpad. Liver tissue sections were taken on day 12 after infection. **(A)** Virus spread in liver tissue. Infection was performed with 10^5^ PFU of mCMV-Δm06^W^, now identified to represent a mixture of correct virus mCMV-Δm06 and a “large deletion” mutant mCMV-Δm06m145-158 that includes deletion of the *m152* gene. 2C-IHC was performed to detect cytoplasmic m152 protein (red staining) specific for mCMV-Δm06 and intranuclear IE1 protein (black staining) expressed by both viruses. (Center panel) overview image showing foci of infection for both viruses in the mixture representing mCMV-Δm06^W^. (Left panel) detail image of cells infected with mCMV-Δm06 identified by red cytoplasmic staining of m152. (Right panel) Detail image of cells infected with mCMV-Δm06m145-158 characterized by absence of red cytoplasmic staining. Counterstaining was performed with hematoxylin. Bar markers: 50 μm. **(B)** vRAP expression-dependent control of liver tissue infection with the indicated viruses on day 12 after adoptive transfer of 10^5^ antiviral CD8 T cells specific for the viral epitope M45-D^b^. 2C-IHC was performed to identify infected cells expressing m152 (red symbols) or lacking m152 (black symbols). (no AT) no adoptive transfer. (AT) adoptive transfer. Symbols represent mice tested individually. Data represent counts of infected liver cells in representative 50 mm^2^ tissue section areas. For further explanation, see the legend to Figure 3.

With this rationale, we also reanalyzed the previously published control of viruses mCMV-WT.BAC, mCMV-Δm06^W^, and mCMV-Δm04m06 in immunocompromised C57BL/6 mice by adoptive transfer of CTL specific for epitope M45-D^b^ (Holtappels et al., 2006). As virus levels are generally lower in tissues of C57BL/6 compared to BALB/c mice, we used the more sensitive 2C-IHC for measuring differential protein expression to distinguish between liver cells infected with the correct mutant mCMV-Δm06 that expresses m152 (black staining of intranuclear IE1 protein and red staining of cytoplasmic m152 protein) and the “large deletion” mutant mCMV-Δm06m145-158 that lacks m152 (only black staining of IE1) in mice infected with the virus mixture mCMV-Δm06^W^ (Figure 4). In accordance with our previous work (Holtappels et al., 2004, 2006, 2009), viruses expressing the negative vRAP m152 were not controlled by M45-D^b^ CTL, whereas again m04 relieved immune evasion by m152 expressed in liver cells infected with the correct mutant mCMV-Δm06.

## Discussion

Focussing first on the technical aspect of our work, we report the worrying observation of a previously unrecognized authenticity failure in a virus that has been widely distributed in the community of CMV immunologists and has led to a number of well-cited publications now to be called into question. One may wonder why an unintended deletion of ≈13 kbp spanning 14 ORFs in mCMV-Δm06^W^ has escaped the standard quality controls employed in the early days of BAC mutagenesis of CMVs. According to the original report on the panel of combinatorial immune evasion gene deletion mutants of mCMV (Wagner et al., 2002), all recombinant BAC plasmids were controlled for correct deletions and for genome integrity by restriction pattern analysis, which reliably did not reveal a ≈13 kbp deletion in recombinant BAC plasmid pΔm06 that was used to reconstitute virus mCMV-Δm06^W^. In addition, correct patterns of combinatorial deletions in the panel of reconstituted viruses were tested by Western blot analysis of immune evasion protein expression (Wagner et al., 2002) and the expected patterns of immune evasion gene deletions were later also confirmed by ISH with specific DNA probes in liver tissue sections of mice infected with the respective viruses (Holtappels et al., 2006). Seen in retrospect, it was a mistake to not have analyzed the reconstituted virion genomic DNA, and that Western blot analysis and ISH were performed only qualitatively. With the knowledge of today, the seemingly correct Western blot and ISH data obtained for mCMV-Δm06^W^ reflected the minority fraction of correct Δm06 mutant present in the now identified virus mixture. Given the structural integrity of pΔm06 (Wagner et al., 2002), the large deletion must have occurred by a recombination event during virus reconstitution and subsequent rounds of propagation in cell culture performed to excise the BAC vector sequence from the BAC-cloned viral genome (Wagner et al., 1999). Suspiciously, it was just pΔm06 that was constructed by using a shuttle plasmid for recombination, whereas all other plasmids in the combinatorial deletion panel were constructed by PCR-based en-passant mutagenesis (Wagner et al., 2002). It remains speculative if the difference in the construction of pΔm06 relates in any way to the later recombination event. To our knowledge, plaque purification was performed for mCMV-Δm06^W^, though even a high clone probability still bears a risk of bi-clonality based on the Poisson distribution statistics that applies to limiting dilution approaches (Lefkovits and Waldmann, 1979). We have in the meantime cloned a new combinatorial panel of immune evasion gene deletion mutants of mCMV and found a phenotype discrepancy to the “Wagner panel” only for the mutants mCMV-Δm06^W^ and mCMV-Δm06^L^. If the case reported here was a rare accident or if there is reason to doubt all mutants published in the early days of CMV mutagenesis, remains unanswered and speculative. Although the previously published conclusion was not falsified, and thus requires no corrigendum, this example teaches us the lesson to always verify the authenticity of viral mutants also beyond the site of the targeted mutation. Clearly, in modern times, NGS of full-length viral genomes is the method of choice. Even NGS, however, would have misled us if performed only at the stage of recombinant BAC plasmids. Thus, the most important lesson, in our view, is to always control genetic authenticity of the full-length virion DNA.

Identification of mCMV-Δm06^W^ as a virus mixture, combined with the fact that we routinely store paraffin-imbedded organs of previously performed animal experiments in a tissue bank, gave us the chance to reanalyze the previous experiments performed by Holtappels et al. (2006) with focus on the correct mutant mCMV-Δm06 identified in liver tissue by 2C-ISH and 2C-IHC. These reanalyses confirmed the previous conclusion of m04 acting as a positive vRAP in antiviral protection, verified for two quite different pMHC-I complexes in two MHC (*H-2*) haplotypes, namely two M45-derived peptides presented by D^d^ and D^b^, respectively.

The question may come up if this function applies to all pMHC-I complexes made up by different antigenic peptides and different peptide-presenting MHC-I alleles. In the original work by Kleijnen et al. (1997) m04/gp34 was described to form “*a complex with folded class I MHC molecules in the ER which is not retained but is transported to the cell surface*.” The authors found an allelic difference in that complexes could be immunoprecipitated from the cell surface of mCMV^Smith^-infected cells with antibodies directed against K^b^, D^b^, L^d^, and D^d^, with K^d^ representing an exception confirming the rule. Accordingly, a more recent structural analysis revealed binding of m04 to a broad range of MHC-I alleles (Berry et al., 2014). Berry and colleagues also considered the high variability of m04 in strains of mCMV (Corbett et al., 2007) and confirmed binding of m04^Smith^, m04^G4^, and m04^W8211^ to L^d^, D^k^, and D^d^. A further layer of complexity is brought in by the recent finding of allelic differences in MATp1-dependent cell surface expression of m04-MHC-I complexes (Železnjak et al., 2019). Actually, there is nothing unusual about allelic differences, since they reflect the evolutionary selection pressure that has led to MHC polymorphism.

The decisive question remained, if MHC-I still presents antigenic peptide in the complex with m04, and, if so, if interactions of pMHC-I with m04 and MATp1 interfere with recognition by the TCR of CD8 T cells. As to the first question, Kleijnen et al. (1997) have already shown TAP-dependence of cell surface expression of m04-MHC-I complexes, so that the MHC-I molecules in the complexes are peptide-loaded. Notably, the effect of MATp1 on cell surface expression was particularly strong for m04-D^d^ complexes, so that our data for M45-D^d^ imply that neither m04 nor MATp1 interaction interfere with recognition by CD8 T cells. Finally, even if exceptions exist depending on MHC-I allele and, possibly, virus strain variance in the m04 sequence, m04 positively regulates antiviral protection because virus control by CD8 T cells is secured redundantly by more than one antigenic peptide presented by any of the MHC-I molecules of a given haplotype [reviewed in Ebert et al. (2012)].

Our data also shed new light on the function of the negative vRAP m06. As the adoptive transfer experiments show, the positive effect of m04 on protection in the presence of just m152 is reduced by m06 co-expressed with m04 and m152 in mCMV-WT.BAC. Thus, while m06 when expressed alone in deletion mutant mCMV-Δm04m152 has only a moderate inhibitory effect on antigen presentation (reviewed in Lemmermann et al., 2012), its main function might be to compete with m04 for MHC-I cargo and thereby reduce the positive effect of m04. Notably, it appears that this competition does not completely abolish the positive effect of m04, as it is indicated by a somewhat better protection against mCMV-WT.BAC compared to mCMV-Δm04m06 expressing only the main negative vRAP m152 (Figures 3, 4). The balanced competitive effects of m04 and m06 also explain the previously poorly understood finding that deletion of just m152 is highly efficient in relieving virus evasion of antiviral CD8 T cells (Holtappels et al., 2004, 2009).

In conclusion, our data give new insights into the interplay between the three vRAPs in virus control by CD8 T cells.

## Data Availability Statement

The datasets generated for this article can be found in European Nucleotide Archive (ENA) using the project number PRJEB38039 (http://www.ebi.ac.uk/ena/data/view/PRJEB38039).

## Ethics Statement

The animal study was reviewed and approved by the ethics committee of the Landesuntersuchungsamt Rheinland-Pfalz, permission number 177-07/021-28.

## Author Contributions

MR and NL were responsible for conception and design of the study, analysis, and interpretation of the data. SB, AF, JP, JS, IG, and TB conducted the work and analyzed the data. MR wrote the first draft of the manuscript. SB, TB, and NL wrote sections of the manuscript. All authors contributed to manuscript revision, read, and approved the submitted version.

## Conflict of Interest

The authors declare that the research was conducted in the absence of any commercial or financial relationships that could be construed as a potential conflict of interest.
